# Comparative analysis of lipid and flavonoid biosynthesis between Pongamia and soybean seeds: genomic, transcriptional, and metabolic perspectives

**DOI:** 10.1186/s13068-024-02538-w

**Published:** 2024-06-24

**Authors:** Chun Liu, Rui Huang, Xingkun Zhao, Ranran Xu, Jianyu Zhang, Xinyong Li, Guodao Liu, Rongshu Dong, Pandao Liu

**Affiliations:** 1 Tropical Crops Genetic Resources Institute & National Key Laboratory for Tropical Crop Breeding, Chinese Academy of Tropical Agricultural Sciences, Haikou, 571101 China; 2 Key Laboratory of Crop Gene Resources and Germplasm Enhancement in Southern China, Ministry of Agriculture and Rual Affairs, Haikou, 571101 China; 3 Key Laboratory of Tropical Crops Germplasm Resources Genetic Improvement and Innovation of Hainan Province, Haikou, 571101 China; 4 School of Tropical Agriculture and Forestry & Sanya Institute Breeding and Multiplication, Hainan University, Haikou, 570228/572025 Sanya China

**Keywords:** Pongamia, Soybean, Multi-omics, Lipids, Flavonoids

## Abstract

**Background:**

Soybean (*Glycine max*) is a vital oil-producing crop. Augmenting oleic acid (OA) levels in soybean oil enhances its oxidative stability and health benefits, representing a key objective in soybean breeding. Pongamia (*Pongamia pinnata*), known for its abundant oil, OA, and flavonoid in the seeds, holds promise as a biofuel and medicinal plant. A comparative analysis of the lipid and flavonoid biosynthesis pathways in Pongamia and soybean seeds would facilitate the assessment of the potential value of Pongamia seeds and advance the genetic improvements of seed traits in both species.

**Results:**

The study employed multi-omics analysis to systematically compare differences in metabolite accumulation and associated biosynthetic genes between Pongamia seeds and soybean seeds at the transcriptional, metabolic, and genomic levels. The results revealed that OA is the predominant free fatty acid in Pongamia seeds, being 8.3 times more abundant than in soybean seeds. Lipidomics unveiled a notably higher accumulation of triacylglycerols (TAGs) in Pongamia seeds compared to soybean seeds, with 23 TAG species containing OA. Subsequently, we identified orthologous groups (OGs) involved in lipid biosynthesis across 25 gene families in the genomes of Pongamia and soybean, and compared the expression levels of these OGs in the seeds of the two species. Among the OGs with expression levels in Pongamia seeds more than twice as high as in soybean seeds, we identified one fatty acyl-ACP thioesterase A (FATA) and two stearoyl-ACP desaturases (SADs), responsible for OA biosynthesis, along with two phospholipid:diacylglycerol acyltransferases (PDATs) and three acyl-CoA:diacylglycerol acyltransferases (DGATs), responsible for TAG biosynthesis. Furthermore, we observed a significantly higher content of the flavonoid formononetin in Pongamia seeds compared to soybean seeds, by over 2000-fold. This difference may be attributed to the tandem duplication expansions of 2,7,4ʹ-trihydroxyisoflavanone 4ʹ-O-methyltransferases (HI4ʹOMTs) in the Pongamia genome, which are responsible for the final step of formononetin biosynthesis, combined with their high expression levels in Pongamia seeds.

**Conclusions:**

This study extends beyond observations made in single-species research by offering novel insights into the molecular basis of differences in lipid and flavonoid biosynthetic pathways between Pongamia and soybean, from a cross-species comparative perspective.

**Supplementary Information:**

The online version contains supplementary material available at 10.1186/s13068-024-02538-w.

## Background

The global energy demand is increasing rapidly, leading to the depletion of non-renewable fossil fuels, such as coal, oil, and natural gas. Bioenergy, considered the fourth-largest energy source worldwide, is gaining traction as an attractive alternative due to its potential as a renewable and sustainable energy source [1, 2]. Unlike finite energy sources, bioenergy can be derived from organic materials that can be replenished relatively quickly, with neutral or even negative carbon emissions. Biofuel plants offer numerous advantages over fossil fuels, including improved soil quality, the promotion of nutrient cycling and carbon fixation, and the generation of large quantities of high-carbon biomass [3, 4]. Therefore, the appropriate selection and application of biofuel plants represent an ideal strategy to rapidly reduce dependence on fossil fuel reserves. Despite the theoretical potential of various oil-bearing plants as biofuel sources, many are unsuitable for industrial-scale production because of their negative implications for food security and cultivated land use. For instance, the expanded use of soybean (*Glycine max*) as a biofuel feedstock could diminish available protein and oil resources for human and animal consumption [5]. Thus, there is a critical need to identify alternative oil-yielding plants that do not conflict with food crops to diversify biofuel source options.

Pongamia (*Pongamia pinnata*) is a versatile leguminous tree found extensively in the tropical and subtropical regions of the Indian subcontinent and Southeast Asia [6]. It is recognized for its diverse applications, serving as a valuable resource for biofuel feedstock, traditional medicine, green manure, timber, animal fodder, biopesticide, and ornamental planting [7]. Pongamia is notable for its capacity to thrive in challenging climatic and soil conditions, showcasing exceptional salt tolerance, resistance to drought, and proficiency in nitrogen fixation [8]. These characteristics make Pongamia suitable for cultivation in diverse environments and low-input agricultural settings. As a prospective biofuel plant, Pongamia produces a substantial yield of non-edible seed oils that are readily extractable and convertible into fuel [9]. Additionally, the resultant fuel derived from Pongamia seeds demonstrates lower levels of sulfur and ash constituents, rendering it environmentally favorable [10]. Notably, the annual oil yield of Pongamia can reach approximately 6,000 L/ha, surpassing the yields reported for many other biofuel plant species [11]. Furthermore, Pongamia seeds contain oil ranging from 35 to 40% of their dry weight, with more than half of it being oleic acid, an ideal fatty acid for producing high-quality biodiesel [12, 13].

In addition to being a biofuel plant, Pongamia also serves as a medicinal plant. Different parts of Pongamia are employed in traditional medicine. For example, flowers are used for addressing bleeding hemorrhoids, seed powder for reducing fever and aiding in the treatment of bronchitis, leaf juice for managing leprosy and flatulence, bark for alleviating coughs and colds, and root extract for treating canker sores, tumors, and skin ailments [8, 14, 15]. One of the primary medicinal components isolated from Pongamia is flavonoids and their derivatives. It has been reported that the oil extracted from Pongamia seeds contains 5–6% flavonoids [16]. Several common simple flavonoids found in leguminous plants, such as kaempferol, quercetin, daidzein, and formononetin, have also been identified in different tissues of Pongamia [17, 18]. Notably, some specific flavonoids have been identified in Pongamia, such as a furanoflavone known as karanjin, which was first isolated from Pongamia seeds in 1925 [19]. The second furanoflavone identified in this species, pongapin, was isolated from the root bark [17]. Glycosylated derivatives of flavonoids have also been isolated from Pongamia. Pongamosides A-D represent the first four glycosidic flavonoids identified in the fruits of this plant [20, 21]. The various flavonoids isolated from Pongamia exhibit diverse biological activities, including antioxidant, antimicrobial, and anti-inflammatory properties [17, 22].

Although various oil, lipid, and flavonoid metabolites have been identified in Pongamia, the genes associated with the biosynthesis of these metabolites remain unclear. Previous studies have attempted to elucidate the mechanisms underlying oil and lipid accumulation in Pongamia seeds using transcriptomic and metabolomic techniques [12, 23, 24]. However, the absence of a high-quality reference genome for Pongamia has constrained comprehensive investigation of this mechanism at the whole-genome level. The recent availability of Pongamia genome sequences has enabled the investigation of genome evolution and the identification of genes involved in important metabolic pathways [25, 26]. Soybean is a major oil crop, and its seeds are important sources of human food, vegetable oil, and bioenergy, having undergone extensive study. Pongamia and soybean belong to the same family (Leguminosae) and subfamily (Papilionoideae) [25, 27]. A comparative study between Pongamia seeds and soybean seeds could be instrumental in evaluating the potential value of Pongamia seeds. In this study, we integrated transcriptome, metabolome, lipidome, and genome analyses to establish a comprehensive multi-omics database for Pongamia seeds and compared them with those of soybean seeds. This analysis aims to investigate the potential molecular mechanisms underlying the high accumulation of lipids and flavonoids in Pongamia seeds, and to identify candidate genes for enhancing oleic acid and active flavonoid content in other oil crops, such as soybean, through genetic engineering.

## Results

### Comparative genomics analysis between Pongamia and soybean

**Fig. 1:**
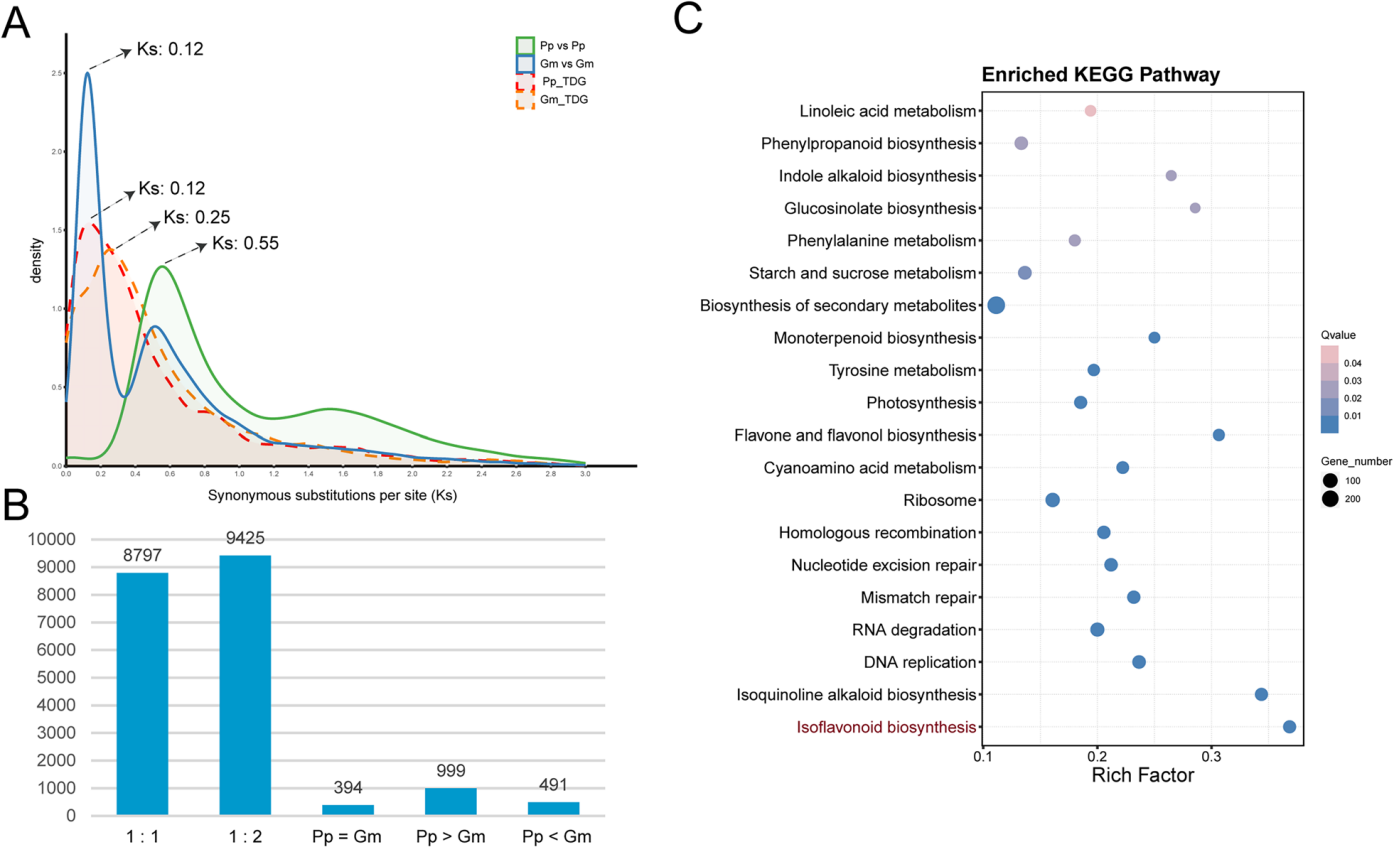
Comparative genomics between Pongamia (Pp) and soybean (Gm). **A** *Ks* distribution of gene paired from intra-species collinearity blocks and tandem duplications of Pongamia and soybean. **B** Comparative statistics of orthologous groups (OGs) between Pongamia and soybean. **C** KEGG enrichment analysis of genes from 999 OGs that have more genes in Pongamia than that in soybean

In this study, a comparative genomics analysis was conducted between Pongamia and soybean to elucidate their commonalities and differences. Intra-specific collinearity analysis in Pongamia and soybean was conducted, and the distribution of synonymous substitutions per synonymous site (Ks) of gene pairs within these collinearity blocks indicated that soybean underwent a recent whole-genome duplication (WGD). Evidence for this includes the Ks peak at approximately 0.12 and an estimated divergence time at around 9.84 million years ago (MYA), which was absent in Pongamia (Fig. 1A). However, both Pongamia and soybean shared a WGD event around 45.08 MYA (Ks peak at about 0.55), reflecting an ancestral WGD in the legume family [27, 28] (Fig. 1A). In addition, 4924 genes (14.04% of the total genes) in Pongamia and 4740 genes (8.53% of the total genes) in soybean were identified as tandem duplication genes (TDGs). The Ks distribution of TDGs revealed a peak at 0.12 (approximately 9.84 MYA) for Pongamia and 0.25 (approximately 20.49 MYA) for soybean (Fig. 1A), suggesting a species-specific tandem duplication event in the Pongamia genome. Furthermore, orthologous groups (OGs) of Pongamia and soybean were also identified, resulting in 20,106 OGs comprising 22,987 in-paralogs from Pongamia and 31,738 in-paralogs from soybean. Although OGs predominantly exhibited a gene ratio of 1:2 (one gene copy in Pongamia to two gene copies in soybean), a noteworthy finding is the presence of 999 OGs, comprising 3246 genes, with a higher copy number in Pongamia compared to soybean (Fig. 1B). The origin of these additional genes in Pongamia was mainly attributed to dispersed (1606 genes) and tandem (814 genes) duplication events within its genome (Additional file 1: Table S1). Kyoto Encyclopedia of Genes and Genomes (KEGG) enrichment analysis of the 3246 genes revealed significant enrichment in "Isoflavonoid biosynthesis" (ko00943), "Isoquinoline alkaloid biosynthesis" (ko00950), and "Flavone and flavonol biosynthesis" (ko00944), etc. (Fig. 1C). It is noteworthy that five genes encoding 2,7,4ʹ-trihydroxyisoflavanone 4ʹ-O-methyltransferases (HI4ʹOMTs), which expanded through tandem duplication in Pongamia, were found within the 999 OGs (Additional file 1: Table S2). HI4ʹOMTs primarily catalyze the synthesis of formononetin and biochanin A in the isoflavonoid biosynthetic pathway [29–31].

### Transcriptome analysis of different tissues of Pongamia

**Fig. 2:**
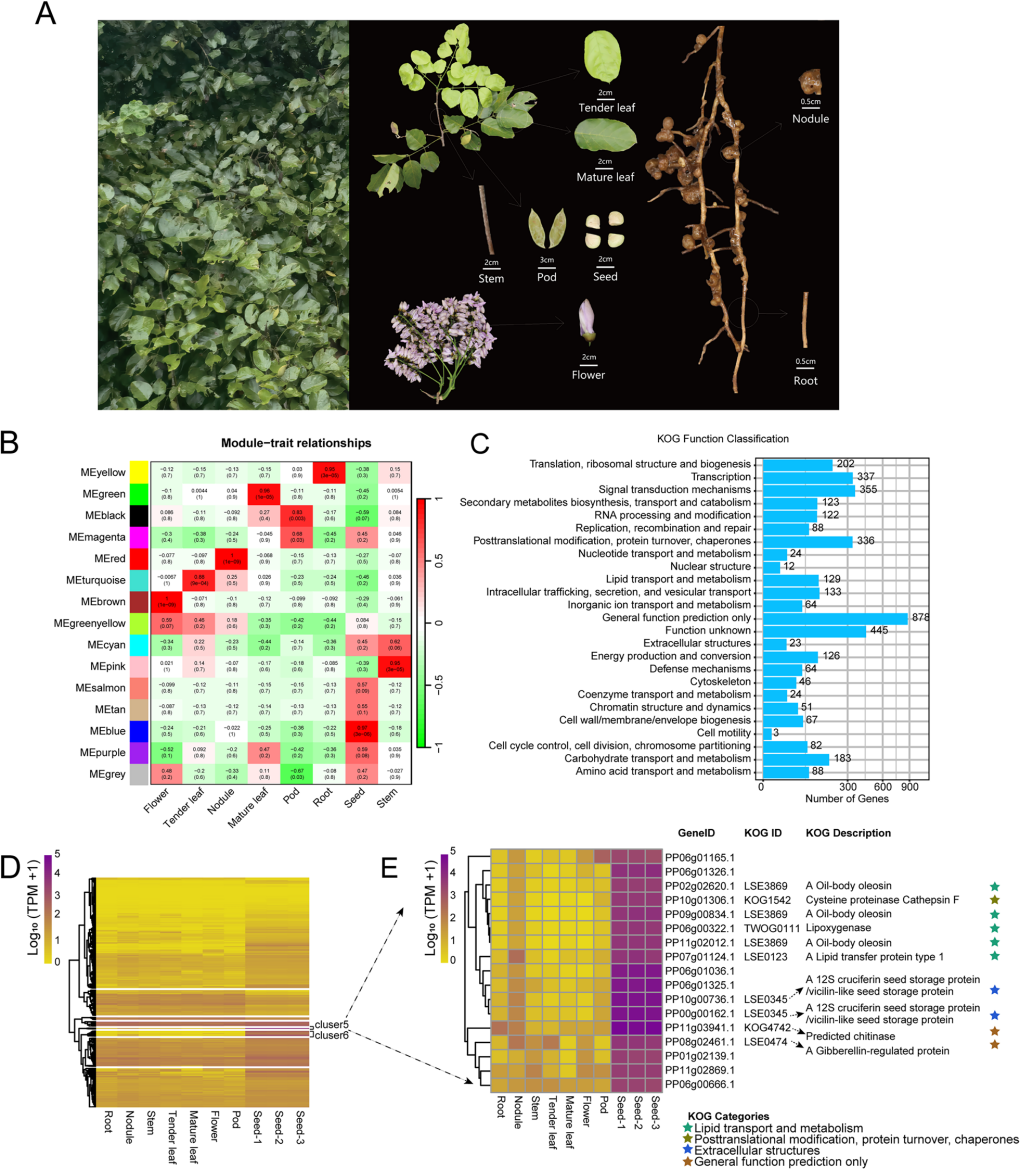
Transcriptome and gene co-expression analyses of eight distinct tissues in Pongamia. **A** Various tissues of Pongamia that used for transcriptome analysis. **B** A gene co-expression module was constructed using WGCNA, showing the correlation coefficient and corresponding *P*-values. **C** KOG categorization of 4724 genes in the blue module, identified as the seed-related module. **D** Expression heatmap of genes in the seed-related module. **E** Expression heatmap of genes specific highly expressed in seeds (cluster5 in **D**)

Ribonucleic acid (RNA) from various tissues of Pongamia, including roots, stems, pods, leaves (both tender and mature leaves), flowers, nodules, and seeds (Fig. 2A), was extracted and sequenced by DNBSEQ-T7 sequencing technology (MGI-Tech, China). Following the exclusion of low-quality reads, a total of 72.18 Gb of clean reads were obtained, with an average of 7.22 Gb per sample (Table 1). Clean reads from different tissues were mapped onto the Pongamia genome [26] and reconstructed transcripts to improve gene annotation. The average genome mapping ratio was 86.88%, with the highest mapping ratio recorded at 91.39% (Additional file 1: Table S3). Additionally, read counts and gene expression levels were calculated using transcripts per kilobase million (TPM), revealing 29,095 genes expressed across Pongamia tissues (Additional file 1: Table S4). Subsequently, a weighted correlation network analysis (WGCNA) was conducted, resulting in the identification of 15 gene co-expression modules (Fig. 2B). Eight of these modules exhibited significant correlations with diverse tissues (*P*-value < 0.01) (Fig. 2B). Among them, the blue module, termed seed-related module, exhibited a strong correlation with seed traits (correlation coefficient = 0.97, *P*-value < 0.001), encompassing 4724 genes and classified into 25 Eukaryotic Orthologous Groups of Proteins (KOG) categories (Fig. 2C). Notably, this module included 129 genes involved in lipid transport and metabolism (Fig. 2C). Furthermore, the constructed gene expression heatmap of these genes showed that two clusters (clusters 5 and 6) were specifically highly expressed in Pongamia seeds (Fig. 2D). Moreover, within cluster 5, there were five genes involved in lipid transport and metabolism, three of which encoded oil-body oleosin genes (Fig. 2E). Thus, we conducted a genome-wide identification of genes encoding lipid-body-membrane proteins, namely oleosins, caleosins, and steroleosins, in the genomes of Pongamia, soybean, and Arabidopsis (Additional file 1: Table S5). As a result, Pongamia genomes contained six genes encoding oleosins, three genes encoding oleosins, and five genes encoding steroleosins (Additional file 1: Table S6). Importantly, nine out of the 14 identified lipid-body-membrane proteins were found to belong to the seed-related module of WGCNA, indicating their preferential expression in Pongamia seeds (Fig. 2D, E, Additional file 2: Fig. S1).

**Table 1:** Statistics of RNA sequencing of different tissues from Pongamia and soybean

Species	Sample name	Abbr	Clean Reads	Clean base (Gb)	Read length	Q20 (%)	Q30 (%)	GC (%)
Pongamia	Root	PP_RT	23,844,449	7.15	150	97.3	92.1	43.5
	Stem	PP_ST	24,127,225	7.24	150	97.3	92.2	43.2
	Pod	PP_PD	24,113,612	7.23	150	97.2	92.0	43.9
	Tender leaf	PP_TL	24,002,472	7.20	150	97.2	91.9	43.5
	Mature leaf	PP_ML	24,039,614	7.21	150	97.4	92.3	43.6
	Flower	PP_FL	24,044,029	7.21	150	97.3	92.1	43.5
	Nodule	PP_ND	24,122,260	7.20	150	97.1	91.6	44.4
	Seed-1	PP_SD-1	24,128,136	7.24	150	97.1	91.7	43.7
	Seed-2	PP_SD-2	24,102,575	7.23	150	97.0	91.5	43.5
	Seed-3	PP_SD-3	24,080,416	7.22	150	97.4	92.4	43.7
Soybean	Seed-1	GM_SD-1	24,087,282	7.23	150	98.1	93.5	45.1
	Seed-2	GM_SD-2	24,037,903	7.21	150	98.1	93.3	45.3
	Seed-3	GM_SD-3	24,126,214	7.24	150	97.9	92.7	45.3

### Comparing differences in metabolite accumulation between Pongamia and soybean seeds via untargeted metabolomics

**Fig. 3:**
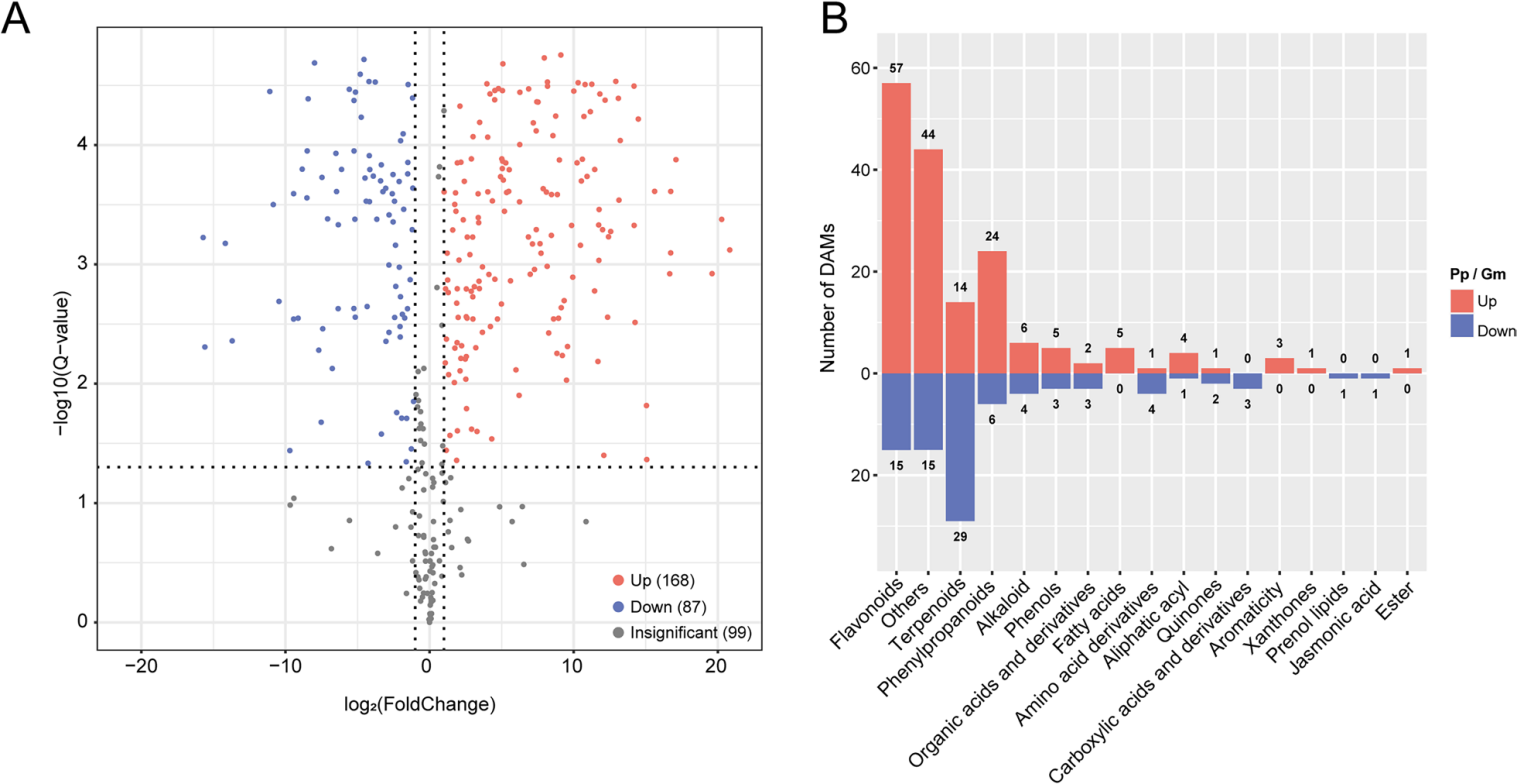
Untargeted metabolomics analysis of Pongamia seeds compared to soybean seeds. **A** Volcano plot of metabolites in Pongamia seeds versus soybean seeds. **B** Classification of differently accumulated metabolites (DAMs) in Pongamia (Pp) seeds versus soybean (Gm) seeds

A total of 354 metabolites were identified in seeds of Pongamia and soybean, with 168 up-regulated and 87 down-regulated in Pongamia seeds when compared to soybean seeds (Fig. 3A, Additional file 1: Table S7). The differentially accumulated metabolites (DAMs) were classified into 17 chemical compound categories, with flavonoids being the most abundant class in Pongamia seeds compared to soybean seeds (Fig. 3B). To explore the pathways involved with these DAMs, we mapped them onto the KEGG pathways. A total of 26 DAMs were mapped onto 36 KEGG pathways, with the highest numbers of DAMs being in the "Metabolic pathways" and "Biosynthesis of secondary metabolites" (Additional file 1: Table S8). More importantly, there were five DAMs involved in "Isoflavonoid biosynthesis", five in "Flavonoid biosynthesis", and four in "Flavone and flavonol biosynthesis" (Additional file 1: Table S8). It is worth mentioning that among the up-regulated flavonoids, the level of karanjin in Pongamia seeds was 1480 times higher than that in soybean seeds (Additional file 1: Table S7). Among the DAMs belonging to isoflavonoids, formononetin exhibited higher accumulation in Pongamia seeds than in soybean seeds (Additional file 1: Table S7; Additional file 3: Fig. S2). Additionally, in Pongamia seeds, DAMs belonging to the class of fatty acids, such as oleic acid (C18:1) and palmitic acid (C16:0), showed higher accumulation compared to those in soybean seeds (Additional file 1: Table S7).

### Differential accumulation of isoflavonoids in Pongamia and soybean seeds

**Fig. 4:**
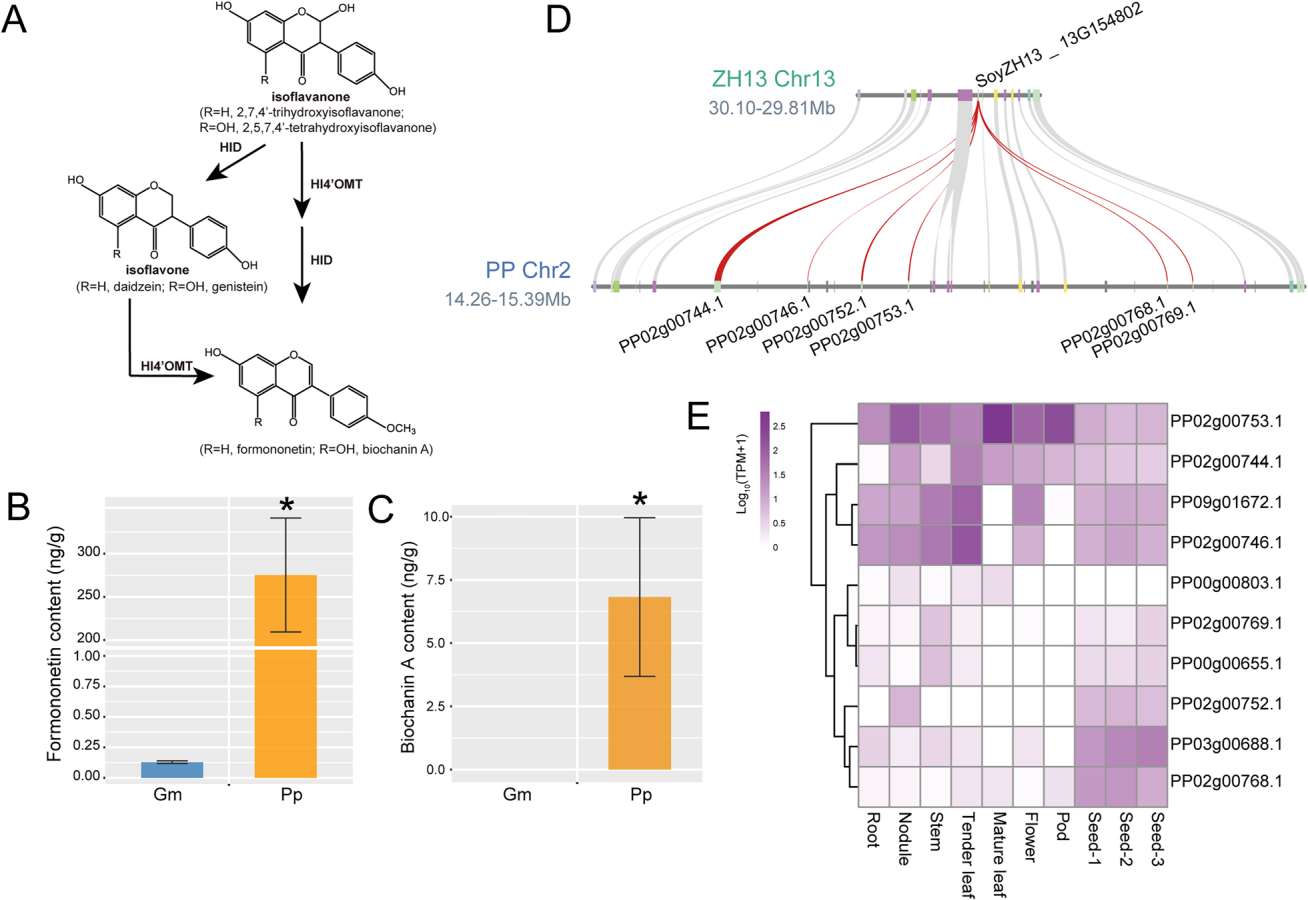
Accumulation of formononetin and biochanin A in Pongamia seeds. **A** HI4'OMT involved in isoflavonoid biosynthesis pathway. **B** Contents of formononetin in the seeds of Pongamia (Pp) and soybean (Gm). **C** Contents of biochanin A in the seeds. Asterisks indicate significant differences between Pongamia seeds and soybean seeds, as determined by Student’s *t*-test (^*^P < 0.05). Biochanin A was not detected in soybean seeds. **D** Microcolinearity of HI4'OMT genes in Pongamia compared to soybean. Red curves indicate the collinearity of HI4'OMTs. **E** Gene expression heatmap of HI4'OMTs in various tissues of Pongamia

In light of the significant enrichment of the "Isoflavonoid biosynthesis" pathway in Pongamia compared to soybean, based on the genes with a higher copy number in Pongamia compared to soybean (Fig. 1C), a quantitative analysis of seven isoflavonoids in both Pongamia and soybean seeds was performed using LC–MS/MS. The results revealed that the contents of five isoflavonoids (i.e., daidzin, daidzein, genistin, genistein, and glycitin) in Pongamia seeds were significantly lower than those in soybean seeds (Additional file 1: Table S9). However, the content of formononetin in Pongamia seeds was 2136-fold higher than that in soybean seeds (Fig. 4B, Additional file 1: Table S9). Additionally, biochanin A was exclusively detected in Pongamia seeds (Fig. 4C, Additional file 1: Table S9). These findings correlated with the tandem duplication expansion of the HI4ʹOMT gene family on Chr2 of the Pongamia genome, which is involved in catalyzing the final step of the biosynthesis for formononetin and biochanin A (Fig. 4A, D). Moreover, gene expression profiles across various tissues revealed high expression levels of HI4'OMT genes, particularly in Pongamia seeds (Fig. 4E, Additional file 1: Table S10).

### Comparative analysis of free fatty acid (FFA) and lipid accumulation differences between Pongamia and soybean seeds

**Fig. 5:**
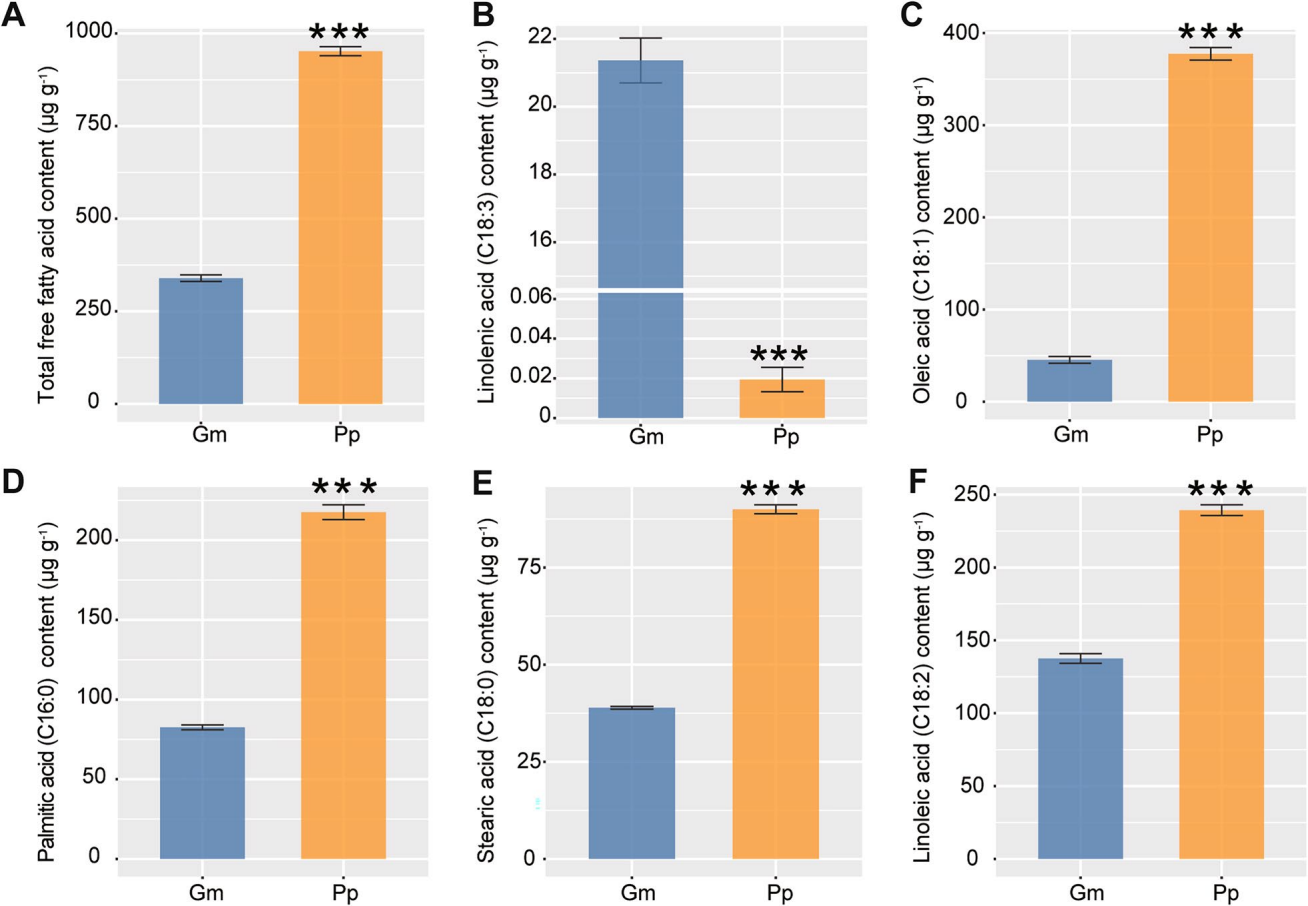
Contents of total free fatty acid (**A**), linolenic acid (**B**), oleic acid (**C**), palmitic acid (**D**), stearic acid (**E**), and linoleic acid (**F**) in the seeds of Pongamia (Pp) and soybean (Gm). Asterisks indicate significant differences between Pongamia seeds and soybean seeds, as determined by Student’s *t*-test (^***^P < 0.001)

The contents of 27 FFAs in both Pongamia and soybean seeds were determined using GC–MS. The results unveiled significantly higher levels of total FFAs in Pongamia seeds compared to soybean seeds (Fig. 5A; Additional file 1: Table S11). Specifically, Pongamia seeds showed higher levels of 16 FFAs compared to soybean seeds (Additional file 1: Table S11). However, one FFA, namely linolenic acid (C18:3), exhibited lower content in Pongamia seeds compared to soybean seeds (Fig. 5B). Notably, oleic acid (C18:1), palmitic acid (C16:0), stearic acid (C18:0), and linoleic acid (C18:2), the four most abundant FFAs in both Pongamia and soybean seeds, exhibited significantly higher levels in Pongamia seeds than in soybean seeds, with respective increases of 8.3-fold, 2.6-fold, 2.3-fold, and 1.7-fold (Fig. 5C–F).

**Fig. 6:**
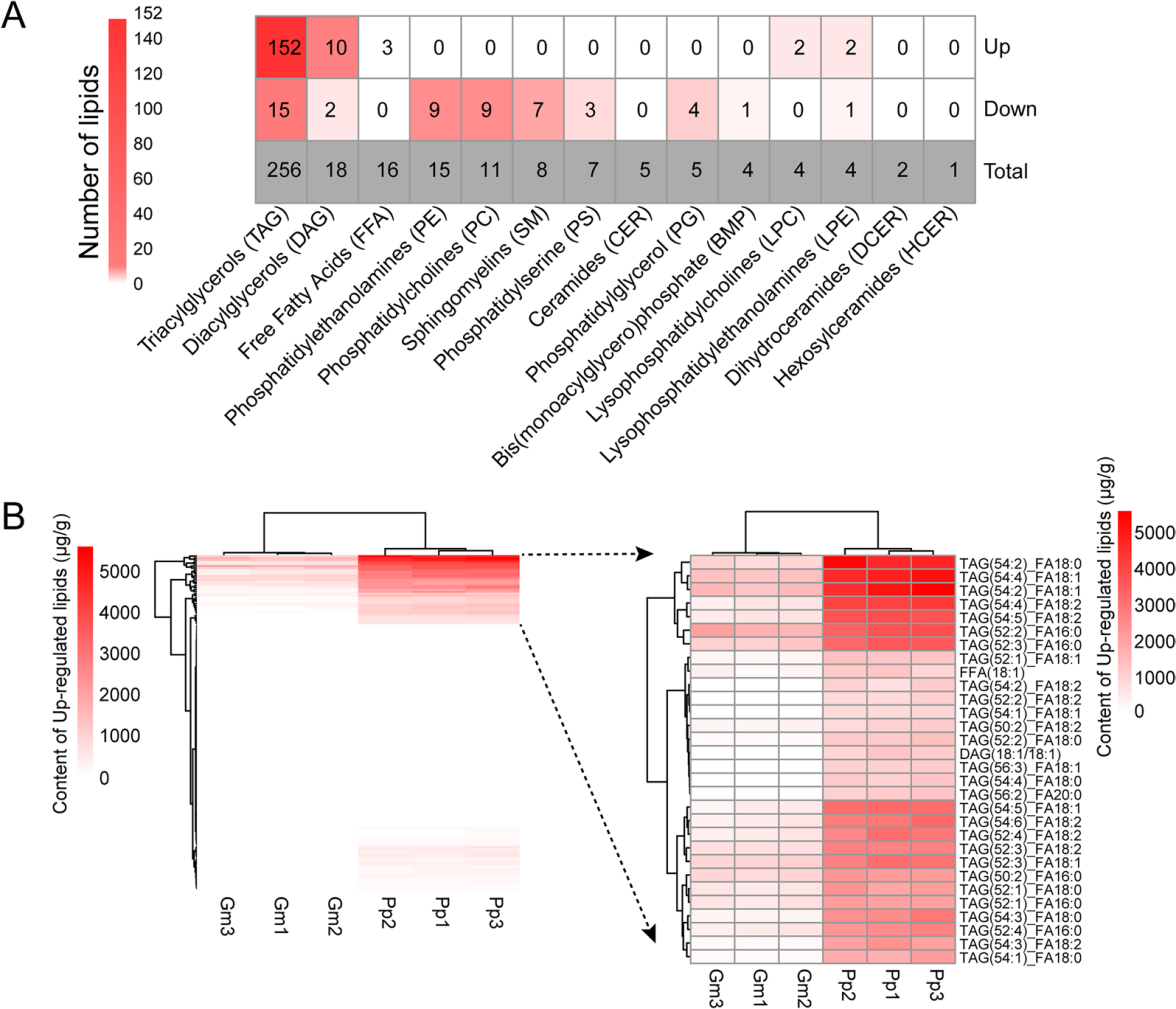
The categories and contents of lipids in seeds of Pongamia and soybean. **A** The categories and numbers of differentially accumulated lipids in Pongamia seeds compared to soybean seeds. **B** Heatmap displaying up-regulated lipids in Pongamia (Pp) seeds compared to soybean (Gm) seeds. The up-regulated lipids with the highest contents are shown on the right

Using lipidomic analysis, a total of 356 lipids across 14 classes were identified in the seeds of Pongamia and soybean (Additional file 1: Table S12), with triacylglycerol (TAG) being the predominant class, comprising 256 individual TAGs (Fig. 6A). Out of these 356 lipids, 220 were identified as differentially accumulated lipids (DALs) between Pongamia and soybean seeds, including 169 up-regulated lipids and 51 down-regulated lipids in Pongamia seeds compared to soybean seeds (Additional file 1: Table S12). Notably, among the 169 up-regulated lipids in Pongamia seeds, 152 belong to the TAG class, and 23 of these TAG species contain oleic acid (C18:1) (Additional file 1: Table S12). Furthermore, heat mapping and cluster analysis were conducted on 169 significantly up-regulated lipids in Pongamia seeds compared to soybean seeds. The results revealed that 30 lipids accumulated the most in Pongamia seeds, comprising 28 TAGs, one diacylglycerol (DAG), and the remaining one being oleic acid (C18:1) (Fig. 6B).

### Comparative analysis of gene families involved in lipid biosynthesis between Pongamia and soybean

**Fig. 7:**
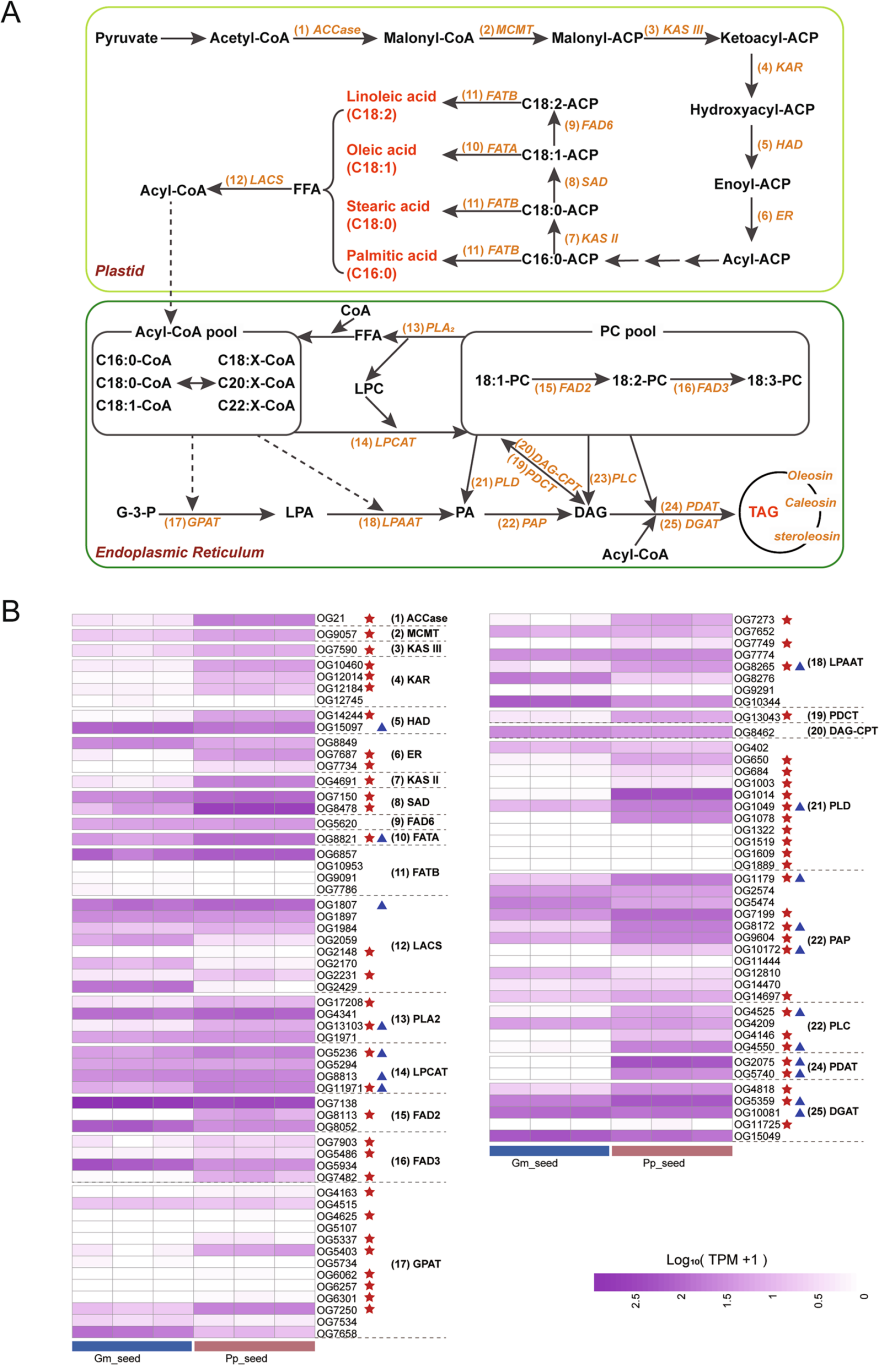
Identification of gene families involved in fatty acid and lipid biosynthesis in Pongamia and soybean. **A** Fatty acid and lipid biosynthesis pathway in plants. **B** Heatmap comparison of expression levels of orthologous groups (OGs) involved in fatty acid and lipid biosynthesis between Pongamia and soybean seeds. The red pentagrams denote OGs in Pongamia seeds with expression levels exceeding those in soybean seeds by more than twofold. The blue triangles indicate OGs belonging to the seed-related module in WGCNA (see Fig. 2)

We identified 25 gene families involved in lipid biosynthesis across the genomes of Pongamia and soybean (Fig. 7A; Additional file 1: Table S13). Consistent with expectations, the soybean genome harbors a higher number of gene copies associated with lipid biosynthesis compared to the Pongamia genome, owing to its recent WGD events, whereas the Pongamia genome has not undergone such events (Additional file 1: Table S14). To compare the gene expression differences between Pongamia and soybean seeds, we employed widely used methods [32, 33] to calculate and compare gene expression levels across species based on OGs. We identified a total of 100 OGs shared by Pongamia and soybean from 25 gene families involved in the biosynthesis of fatty acids and lipids (Additional file 1: Table S15). Among these OGs, 59 from 22 gene families exhibited higher expression levels in Pongamia seeds compared to soybean seeds, exceeding a threshold of twofold difference (Fig. 7B). This subset includes one fatty acyl-ACP thioesterase A (FATA), one ketoacyl-ACP synthase II (KAS II), and two stearoyl-ACP desaturases (SADs), which are involved in oleic acid biosynthesis; two phospholipid:diacylglycerol acyltransferases (PDATs) and three acyl-CoA:diacylglycerol acyltransferases (DGATs), which are involved in TAG biosynthesis; and two phospholipase A2 (PLA2s), three non-specific phospholipase Cs (PLCs), and ten phospholipase Ds (PLDs), which are involved in phosphatidylcholine (PC) catabolism (Fig. 7B).

## Discussions

With continuous advancements in genomics, transcriptomics, proteomics, metabolomics, and bioinformatics technologies, multi-omics approaches have been widely employed to elucidate the molecular genetic basis of key breeding traits, such as yield, quality, and stress adaptation, in legume crops like soybean, cowpea (*Vigna unguiculata*), chickpea (*Cicer arietinum*), faba bean (*Vicia faba*), stylo (*Stylosanthes guianensis*), and pigeonpea (*Cajanus cajan*) [34–37]. In this study, we integrated multi-omics approaches to compare the differences in metabolite accumulation between Pongamia and soybean seeds. Results from both untargeted metabolomics and lipidomics analyses revealed that the levels of oleic acid (C18:1) and linoleic acid (C18:2) were significantly higher in Pongamia seeds compared to soybean seeds (Additional file 1: Tables S7 and S12). This finding is consistent with previous reports indicating that Pongamia seeds are rich in oleic acid and linoleic acid [7, 38]. Subsequent absolute quantification of FFAs further confirmed that oleic acid (C18:1) is the predominant FFA (constituting 40% of total FFAs) in Pongamia seeds, with a content 8.3 times higher than that found in soybean seeds (Fig. 5; Additional file 1: Table S11). Additionally, three other major accumulated FFAs, linoleic acid (C18:2), palmitic acid (C16:0), and stearic acid (C18:0), had significantly higher levels in Pongamia seeds compared to soybean seeds (Fig. 5). To elucidate the potential mechanisms underlying the differences in FFA accumulation between Pongamia and soybean seeds, we employed an OG-based method to compare the expression levels of genes involved in lipid biosynthesis in the seeds of these two species. The results showed that two OGs belonging to SADs and one OG belonging to FATA had expression levels in Pongamia seeds more than twice as high as those in soybean seeds (Fig. 7B). Furthermore, this FATA belongs to the seed-related module in WGCNA, indicating its high expression in Pongamia seeds (Figs. 2B, 7B). SAD and FATA are two crucial enzymes directly involved in oleic acid biosynthesis. SAD catalyzes the conversion of C18:0-ACP to C18:1-ACP, followed by FATA hydrolyzing C18:1-ACP to yield oleic acid (C18:1) [39]. These findings suggest that FATA and SAD exhibit high expression levels in Pongamia seeds, potentially contributing to the accumulation of oleic acid in the seeds.

Apart from FATA, another type of fatty acyl-ACP thioesterase (FAT) is FATB. Unlike FATA, which exhibits high specificity towards oleoyl (C18:1)-ACP (unsaturated acyl-ACPs), FATB demonstrates higher affinity for saturated acyl-ACPs, such as palmitoyl (C16:0)-ACP and stearyl (C18:0)-ACP [40]. In this study, none of the four OGs belonging to FATBs exhibited expression levels in Pongamia seeds that surpassed those in soybean seeds by more than two-fold (Fig. 7B). However, we identified a specific FATB (i.e., PP_uniq94) unique to Pongamia, which showed high expression levels in Pongamia seeds (Additional file 1: Table S15). The elevated levels of palmitic acid and stearic acid in Pongamia seeds may be associated with the specific FATB, yet further validation of its functionality is required. Notably, one OG belonging to fatty acid desaturase 2 (FAD2) and two OGs belonging to PLA2 are expressed at levels more than twice as high in Pongamia seeds compared to soybean seeds (Fig. 7B). In soybean, rapeseed (*Brassica napus*), and peanut (*Arachis hypogaea*), FAD2 is responsible for converting oleic acid (C18:1) into linoleic acid (C18:2) [39, 41]. PLA2 can hydrolyze PC to generate lysophosphatidylcholine (LPC) and FFA [42]. These findings suggest that the high contents of linoleic acid, palmitic acid, and stearic acid in Pongamia seeds may be attributed to the high expression levels of FATB, FAD2, and PLA2.

TAGs serve as the predominant lipid form for carbon and energy storage in plant seeds, accumulating in large quantities [43]. In this study, lipidomic analysis identified 167 TAGs as DALs between Pongamia and soybean seeds, of which 152 TAGs showed significantly higher accumulation in Pongamia seeds (Fig. 6A). It is noteworthy that among the 28 most abundant TAGs in Pongamia seeds, 27 contain at least one of the four fatty acids: oleic acid (C18:1), palmitic acid (C16:0), stearic acid (C18:0), or linoleic acid (C18:2), all of which were found to be significantly higher in Pongamia seeds compared to soybean seeds in this study (Figs. 5, 6B). In plants, two distinct gene families, namely DGAT and PDAT, have been documented to be responsible for the final step of TAG biosynthesis. DGAT utilizes acyl-CoA and DAG as substrates, while PDAT utilizes PC and DAG as substrates [43, 44]. In this study, among the OGs with expression levels in Pongamia seeds more than two times higher than in soybean seeds, three DGATs and two PDATs were identified (Fig. 7B). In plants, two phospholipase families, namely PLC and PLD, participate in the hydrolysis of PC pools to produce DAGs, a precursor in the biosynthesis of TAGs [42]. We found that the expression levels of three OGs belonging to PLCs and ten OGs belonging to PLDs in Pongamia seeds are more than twice those in soybean seeds (Fig. 7B). Consistent with these findings, lipidomics analysis identified nine PCs as DALs, all of which showed significantly lower accumulation in Pongamia seeds compared to soybean seeds (Fig. 6A). These findings suggest that the high expression of PLCs, PLDs, DGATs, and PDATs in Pongamia seeds may facilitate the degradation of PC pools, leading to the accumulation of TAGs.

As a medicinal plant, Pongamia contains numerous flavonoids belonging to the subclasses of flavanones, flavones, flavonols, isoflavonoids, and chalcones, which have been identified in its various tissues [17, 20]. Among them, karanjin is a medicinally valuable flavone isolated from Pongamia seeds. Typically, Pongamia seed extracts yield oil containing 2–4% karanjin [7]. In this study, untargeted metabolomics analysis identified karanjin in Pongamia seeds, with an accumulation level 1,480 times higher than that found in soybean seeds (Additional file 1: Table S7). Additionally, the untargeted metabolomic analysis also revealed a significantly higher accumulation of an isoflavonoid, formononetin, in Pongamia seeds compared to soybean seeds (Additional file 1: Table S7). Subsequently, targeted quantitative analysis showed that the content of formononetin in Pongamia seeds was 2136 times higher than that in soybean seeds (Fig. 4B). In line with the accumulation of formononetin, we observed that HI4'OMT genes, responsible for catalyzing the conversion of daidzein to formononetin [31], have expanded through tandem duplication and are accompanied by high expression levels in Pongamia seeds (Fig. 4D, E). Additionally, untargeted metabolomics analysis and absolute quantification of isoflavonoids both revealed that the level of daidzein, as a substrate for HI4'OMT, was significantly lower in Pongamia seeds compared to soybean seeds (Additional file 1: Table S7 and S9). Similar findings have been reported in pigeonpea, where genes involved in the flavonoid biosynthesis pathway undergo expansion by tandem duplications, contributing to the accumulation of flavonoids in pigeonpea [45]. Based on these findings, it is suggested that the tandem duplicated genes of HI4'OMTs in Pongamia may enhance formononetin biosynthesis by utilizing daidzein as a substrate.

## Conclusion

This study utilized multi-omics analyses to compare the differences in lipid and flavonoid biosynthetic pathways between Pongamia and soybean seeds. We identified OGs shared between Pongamia and soybean, originating from 25 gene families associated with lipid metabolism. Among these OGs, we observed that the expression levels of key genes involved in oleic acid biosynthesis (i.e., FATA and SAD) and TAG biosynthesis (i.e., DGAT and PDAT) in Pongamia seeds were more than twice those in soybean seeds. This could be a key factor contributing to significantly higher levels of oleic acids and TAGs in Pongamia seeds compared to soybean seeds. Furthermore, we observed that the HI4'OMT genes, responsible for formononetin biosynthesis, have expanded through tandem duplication events in the Pongamia genome. These genes exhibited high expression levels in the seeds, which may account for the accumulation of formononetin in Pongamia seeds surpassing that in soybean seeds. The findings of this study will serve as a reference for future research aimed at enhancing and improving the relevant seed traits of Pongamia and soybean.

## Materials and methods

### Sample preparation and RNA sequencing

In 2022, various samples of Pongamia, including roots, stems, pods, tender leaves, mature leaves, flowers, nodules, and seeds, as well as seed samples of the soybean cultivar 'Zhonghuang 13 (ZH13)', were collected at the germplasm nursery of the Chinese Academy of Tropical Agricultural Sciences (CATAS) in Hainan, China. RNA from each tissue was extracted and mRNA with poly A tail was enriched using oligo dT beads. Finally, RNAs were sequenced using a DNBSEQ-T7 sequencer (MGI-Tech, China). Quality control measure for raw sequencing data was conducted using SOAPnuke [46] with specific parameters (–n 0.01 –l 20 –q 0.4 –adaMR 0.25 –ada_trim –polyX 50 –minReadLen 150) to ensure the reliability and accuracy of the resulting data. Three biological replicates were used for the seed samples.

### Genomics data retrieval and transcriptome analysis

The genome sequences of Pongamia, produced through PacBio SMRT and high-throughput chromosome conformation capture (Hi-C), were retrieved from the National Genomics Data Center (NGDC) under the accession number of GWHBCKS00000000 [26]. Benchmarking Universal Single-Copy Orthologs (BUSCO, v5.4.3) analysis was performed to assess the completeness of the genome of Pongamia, resulting in completeness of 97.4%, 4.1% of which were complete and single-copy BUSCOs. Clean reads from different tissues were aligned to the Pongamia genome by HiSat2 (v2.1.0) and transcripts were reconstructed using StringTie (v2.0) [47], yielding a total of 27,605 transcripts. To improve the annotation of protein-coding genes, the reconstructed transcripts, as well as genes predicted through De novo by Augustus (v3.3.1) and SNAP (v2006-07-–28) and homology by GeneWise (v2.4.1) analysis were integrated using EVM (v1.1.1). The plant species selected for homology prediction included *Arabidopsis thaliana*, *Glycine max*, *Medicago truncatula*, *Lupinus albus*, *Phaseolus vulgaris* and *Vitis vinifera*. Ultimately, 35,072 non-redundant protein-coding genes were identified, with 96.8% exhibiting complete BUSCOs (92.8% single-copy and 4.0% duplicated BUSCOs). Functional annotation of protein-coding genes was conducted by assigning these genes to the NCBI non-redundant protein database (NR), KOG, and KEGG. Protein sequences from P. pinnata, G. max, and A. thaliana were aligned against NR, KOG, and KEGG using Diamond software (v2.1.1) [48] with e-value threshold set at 1e−5. Subsequently, the best hit for each gene was retained for further analysis. To ensure consistency in subsequent analyses, the longest transcript was chosen as the representative for genes with multiple transcripts. Gene read counts were determined using featureCounts (v1.6.5), and TPM calculations were performed using a custom Perl script. Gene co-expression modules and tissue correlation analysis were conducted using WGCNA [49] (v1.70–3) under the R platform (v4.2.0). Heatmaps depicting gene expression and metabolite content were generated using pheatmap (v1.0.12). To compare gene expression between Pongamia and soybean seeds, we collected soybean seeds and performed transcriptome sequencing with three biological replicates. The clean reads from soybean seeds were mapped to the soybean genome (Zhonghuang 13, ZH13_v2.0) [50]. The read count and TPM of each soybean gene were calculated using the same method as that adopted for Pongamia.

### Genome comparison analysis of Pongamia and soybean

Genomic collinearity between Pongamia and soybean was analyzed using MCScanX based on the results of alignment of protein sequences. The synonymous substitutions per synonymous site (Ks) value of gene paired within the collinearity block was calculated using ParaAT (v2.0) employing the NG method [51]. Similarly, The Ks value for gene paired of TDG was calculated using the same method. The estimated divergence time of each gene pair was calculated by the formula T = Ks/2r, and a neutral substitution rate of 6.1 × 10^−9^ Ks year^−1^ was adopted [52]. Identifying orthologous groups (OGs) across different species and comparing gene expression based on these OGs are widely used methods in plant research [32, 33, 53]. In our study, we identified OGs between Pongamia and soybean using InParanoid (v5.0). The genes shared by soybean and Pongamia in the OGs are named "OG", followed by a number. OGs specific to Pongamia are defined as "PP_uniq" followed by a number, while those specific to soybean are defined as "GM_uniq" followed by a number. Furthermore, we conducted a comparison of gene expression levels between Pongamia and soybean based on the identified OGs.

### Untargeted metabolomics analysis

Biotree Biomedical Technology Co., Ltd., an independent commercial laboratory situated in Shanghai, China, conducted and analyzed untargeted metabolomics. In brief, approximately 100 mg of each crushed sample of Pongamia and soybean seeds were individually mixed with 500 µL of an extraction solution. This solution consisted of an 80% methanol/water (v/v) mixture, with 1 µg/mL of 2-chloro-l-phenylalanine as the internal standard. After a 30-s vortex, the mixture underwent 1-h sonication in an ice-water bath to extract metabolites. Following a 1-h incubation at − 40 °C, the mixture was centrifuged at 13,800*g* for 15 min at 4 °C. The resulting supernatant was filtered through a 0.22 µm microporous membrane and used for LC–MS/MS analysis. A quality control sample was prepared by combining equal aliquots of the supernatants from all samples. LC–MS/MS analysis employed a Vanquish UHPLC system (Thermo Fisher Scientific, USA) with a UPLC BEH C_18_ column (2.1 × 100 mm, 1.7 μm particle size; Waters, USA) coupled to a Q Exactive Focus mass spectrometer (Thermo Fisher Scientific, USA) equipped with Xcalibur software. The conditions for the UHPLC and MS analyzed were as described previously [54]. Metabolite identification was performed based on an in-house metabolite database provided by Shanghai Biotree Biotech Co., Ltd. Metabolites that exhibited a fold change of ≥ 2 or ≤ 0.5 in relative abundance between Pongamia seeds and soybean seeds, with an adjusted *P*-value (*P*_adj_) < 0.05, were identified as differentially accumulated metabolites (DAMs). Furthermore, KEGG pathway mapping of DAMs were performed based on the KEGG database with the KEGG organisms selected as soybean.

### Determination of flavonoids

The quantitative analysis of flavonoids was performed using a targeted metabolomics service provided by Shanghai Applied Protein Technology Co. Ltd. (China), following the methodology previously described by Mi et al. [55]. In brief, approximately 100 mg of each crushed sample of Pongamia and soybean seeds was individually combined and homogenized with 500 μL of a 70% methanol/water (v/v) solution using ultrasonic-assisted extraction for 30 min. The resulting mixture underwent centrifugation at 14,000*g* for 20 min at 4 °C. The obtained supernatant was used for LC–MS/MS analysis. The LC–MS/MS system comprised an ACQUITY UPLC I-Class system (Waters, USA), a QTRAP 5500 mass spectrometer (AB SCIEX, USA) with an electrospray ionization source (ESI), and an ACQUITY UPLC BEH C_18_ column (2.1 × 150 mm, 1.7 μm particle size; Waters, USA). The UPLC and MS conditions were conducted according to the protocol outlined by Mi et al. [55]. A set of standard solutions with varying gradient concentrations was prepared and analyzed using LC–MS/MS to quantify signal intensities. These intensities were then plotted on the *y*-axis against the corresponding concentration values on the *x*-axis to generate a standard curve. The curve was then used to calculate the concentrations of test samples by substituting their signal intensity values. The study utilized standard substances, including seven isoflavonoids (formononetin, daidzin, daidzein, genistin, genistein, glycitin, and biochanin A), procured from Shanghai Yuanye Bio-Technology Co., Ltd. (China).

### Determination of FFAs

Biotree Biomedical Technology Co., Ltd. (Shanghai, China) conducted a quantitative analysis of FFAs in crushed samples from Pongamia and soybean seeds. Approximately 25 mg samples were placed in 2 mL centrifuge tubes and extracted with a 2:3 (v/v) mixture of isopropanol:n-hexane (500 µL), which contained 0.2 mg/L of stearic-d35 acid as the internal standard. The samples were homogenized in a ball mill for 4 min at 40 Hz, followed by sonication for 30 min in an ice water bath. After centrifugation for 15 min at 13,800*g* and 4 °C, the supernatant was transferred to a fresh 1.5 mL centrifuge tube. A 400 μL portion of the supernatant was freeze-dried using a centrifuge concentrator. Subsequently, a 1:2 (v/v) mixture of methanol and (trimethylsilyl)diazomethane (250 µL) was added, vortex-mixed for 10 s, and left at room temperature for 30 min. The sample was then subjected to nitrogen blow-drying. Following this, 160 μL of n-hexane was added for redissolution, and the solution was centrifuged for 1 min at 13,800*g*. The resulting supernatant was subjected to subsequent analysis using a GC–MS system. This system consisted of an Agilent 7890B gas chromatograph (Agilent Technologies, USA) equipped with a DB-FastFAME capillary column and an Agilent 5977B mass spectrometer (Agilent Technologies, USA). The GC and MS conditions were conducted according to the protocol described previously [56]. The total FFA content is calculated by summing the amounts of all detected FFAs.

### Lipidomics analysis

Biotree Biomedical Technology Co., Ltd. (Shanghai, China) conducted a lipidomics analysis on Pongamia and soybean seeds. In brief, a 25 mg crushed sample was mixed with 400 µL of ultrapure water and subjected to vortexing for 60 s. The mixture was then homogenized at 45 Hz for 4 min and sonicated for 5 min in an ice bath. This process was repeated three times consecutively. Following this, a 10 µL homogenate was combined with 190 µL water, followed by the addition of 480 µL of extract solution comprising methyl *tert*-butyl ether (MTBE) and methanol in a 5:1 (v/v) ratio, containing an internal standard. After centrifugation at 845 g for 15 min at 4 °C, 250 µL of the resulting supernatant was transferred to a centrifuge tube and subjected to vacuum concentration. The dried samples were resuspended in a 2:1 (v/v) mixture of dichloromethane:methanol (100 µL), followed by vortexing for 30 s and sonication for 10 min in an ice-water bath. The resulting solution was centrifuged at 13,523 g for 15 min at 4 °C, and the supernatant was then used for subsequent analysis with an LC–MS/MS system. This system comprised a SCIEX ExionLC series UHPLC System (AB Sciex, USA), an ACQUITY UPLC HSS T3 column (2.1 × 100 mm, 1.8 µm particle size; Waters, USA), and a SCIEX QTRAP 6500 + mass spectrometer (AB Sciex, USA). The experimental parameters for UHPLC and MS, along with procedures for lipid identification and data processing, were carried out as described previously [57]. Lipids that exhibited a fold change of ≥ 2 or ≤ 0.5 in relative abundance between Pongamia seeds and soybean seeds, with an adjusted *P*-value (*P*adj) < 0.05, were identified as differentially accumulated lipids (DALs), following the previous method [58].

### Identification of genes involved in fatty acid and lipid biosynthesis

The identification of genes involved in fatty acid and lipid synthesis in Pongamia and soybean was performed using the method reported by Yang et al. [59], with some modifications. In brief, Arabidopsis genes involved in the fatty acid and lipid biosynthesis pathways were retrieved from ARALIP (http://aralip.plantbiology.msu.edu/pathways/pathways). Next, the protein sequences of Arabidopsis served as the database against which the protein sequences of Pongamia and soybean were compared using Diamond Blastp (v2.1.1). Finally, genes with sequence coverage greater than 50% and similarity greater than 35% were considered as candidate genes.

### Statistical analysis

In this study, we conducted a comparative analysis of the seeds of Pongamia and soybean. The primary statistical method employed was Student’s *t*-test and log_2_-transformed fold change. The false discovery rate (FDR) was calculated using the *P*.adjust function and the 'BH' (Benjamini-Hochberg) algorithm under the R platform (v4.0.2). Genes from the orthologous groups were categorized into KEGG pathways, and an enrichment analysis of these pathways was conducted using the 'phyper' function in R software (v4.0.2). Subsequently, the *P*.adjust function in R, applying the 'BH' algorithm, was used for FDR calculation. Pathways with an FDR < 0.05 were identified as significantly enriched.

### Supplementary Information

Additional file 1: Table S1. The origin of additional genes from 999 OGs that were more copy number in Pongamia than that in soybean. Table S2. KEGG classification of 999 OGs that were more copy number in Pongamia than that in soybean. Table S3. Mapping ratio of RNA from various tissues of Pongamia. Table S4. Gene expression profiles in various tissues of Pongamia. Table S5. Identification of three gene families encoding lipid-body-membrane proteins (oleosin, caleosin, and steroleosin) in *P. pinnata*, *G. max*, and *A. thaliana*. Table S6. Statistics of members from three gene families encoding lipid-body-membrane proteins (oleosin, caleosin, and steroleosin) in *P. pinnata* and *G. max*. Table S7. Differentially accumulated metabolites in Pongamia seeds compared to soybean seeds using untargeted metabolomics analysis. Table S8. KEGG pathway mapping of differentially accumulated metabolites identified through untargeted metabolomics analysis. Table S9. Targeted determination and absolute quantification of flavonoids in seeds of Pongamia and soybean. Table S10. Gene expression of HI4'OMTs in different tissues of Pongamia. Table S11. Targeted determination and quantification of FFAs in seeds of Pongamia and soybean. Table S12. Lipidomic analysis of Pongamia and soybean seeds. Table S13. Identification of genes related to fatty acid and lipid biosynthesis in the genomes of *P. pinnata* and *G. max.* Table S14. Statistics of genes related to fatty acid and lipid biosynthesis in *P. pinnata*, *G. max*, and *A. thaliana.* Table S15. Expression profiles of OGs involved in fatty acid and lipid biosynthesis in *P. pinnata* and *G. max*.

Additional file 2: Figure S1. Expression heatmap of three gene families encoding lipid-body-membrane proteins (oleosin, caleosin, and steroleosin), with nine members found to belong to the seed-related module of WGCNA.

Additional file 3: Figure S2. DAMs belonging to the isoflavonoid biosynthesis pathway. The significant up-regulation and down-regulation of isoflavonoids in Pongamia seeds, as compared to soybean seeds, are highlighted with red and blue dots, respectively.

## Data Availability

Data supporting the findings of this work are available within the paper and associated supplementary data. Genome sequences of Pongamia were retrieved from National Genomics Data Center (NGDC) with accession number of GWHBCKS00000000 (He et al., 2022) [26]. The updated gene annotation and transcriptome data from different tissues of Pongamia, as well as from seeds of soybean, have been submitted to the National Genomics Data Center (NGDC) under PRJCA018629.

## Abbreviations

ACCase: Acetyl-CoA carboxylase
BUSCO: Benchmarking Universal Single-Copy Orthologs
DAG-CPT: Diacylglycerol cholinephosphotransferase
DAG: Diacylglycerol
DAL: Differentially accumulated lipid
DAM: Differentially accumulated metabolite
DGAT: Acyl-CoA:diacylglycerol acyltransferase
ER: Enoyl-ACP reductase
FAD2: Fatty acid desaturase 2
FAD3: Fatty acid desaturase 3
FAD6: Fatty acid desaturase 6
FATA: Fatty acyl-ACP thioesterase A
FATB: Fatty acyl-ACP thioesterase B
FDR: False discovery rate
FFA: Free fatty acid
GPAT: Glycerol-3-phosphate acyltransferase
HAD: Hydroxyacyl-ACP dehydrase
HI4'OMT: 2,7,4'-Trihydroxyisoflavanone 4'-O-methyltransferase
Hi-C: High-throughput chromosome conformation capture
KAR: Ketoacyl-ACP reductase
KAS III: Ketoacyl-ACP synthase III
KAS II: Ketoacyl-ACP synthase II
KEGG: Kyoto encyclopedia of genes and genomes
KOG: Eukaryotic orthologous groups of proteins
Ks: Synonymous substitutions per synonymous site
LACS: Long-chain acyl-CoA synthetase
LPAAT: 1-Acylglycerol-3-phosphate acyltransferase
LPCAT: Lysophosphatidylcholine acyltransferase
MCMT: Malonyl-CoA: ACP malonyltransferase
MYA: Million years ago
NR: NCBI non-redundant protein database
OA: Oleic acid
OG: Orthologous group
PAP: Phosphatidate phosphatase
PC: Phosphatidylcholine
PDAT: Phospholipid:diacylglycerol acyltransferase
PDCT: Phosphatidylcholine:diacylglycerol cholinephosphotransferase
PLA2: Phospholipase A2
PLC: Non-specific phospholipase C
PLD: Phospholipase D
SAD: Stearoyl-ACP desaturase
TAG: Triacylglycerol
TDG: Tandem duplication gene
TPM: Transcripts per kilobase million
WGCNA: Weighted correlation network analysis
WGD: Whole-genome duplication
