# Supplementary figures and images for: Comparative analysis of lipid and flavonoid biosynthesis between Pongamia and soybean seeds: genomic, transcriptional, and metabolic perspectives
Source: Biotechnol Biofuels Bioprod. 2024 Jun 24;17:86. doi: 10.1186/s13068-024-02538-w (PMC11197198; doi:10.1186/s13068-024-02538-w)

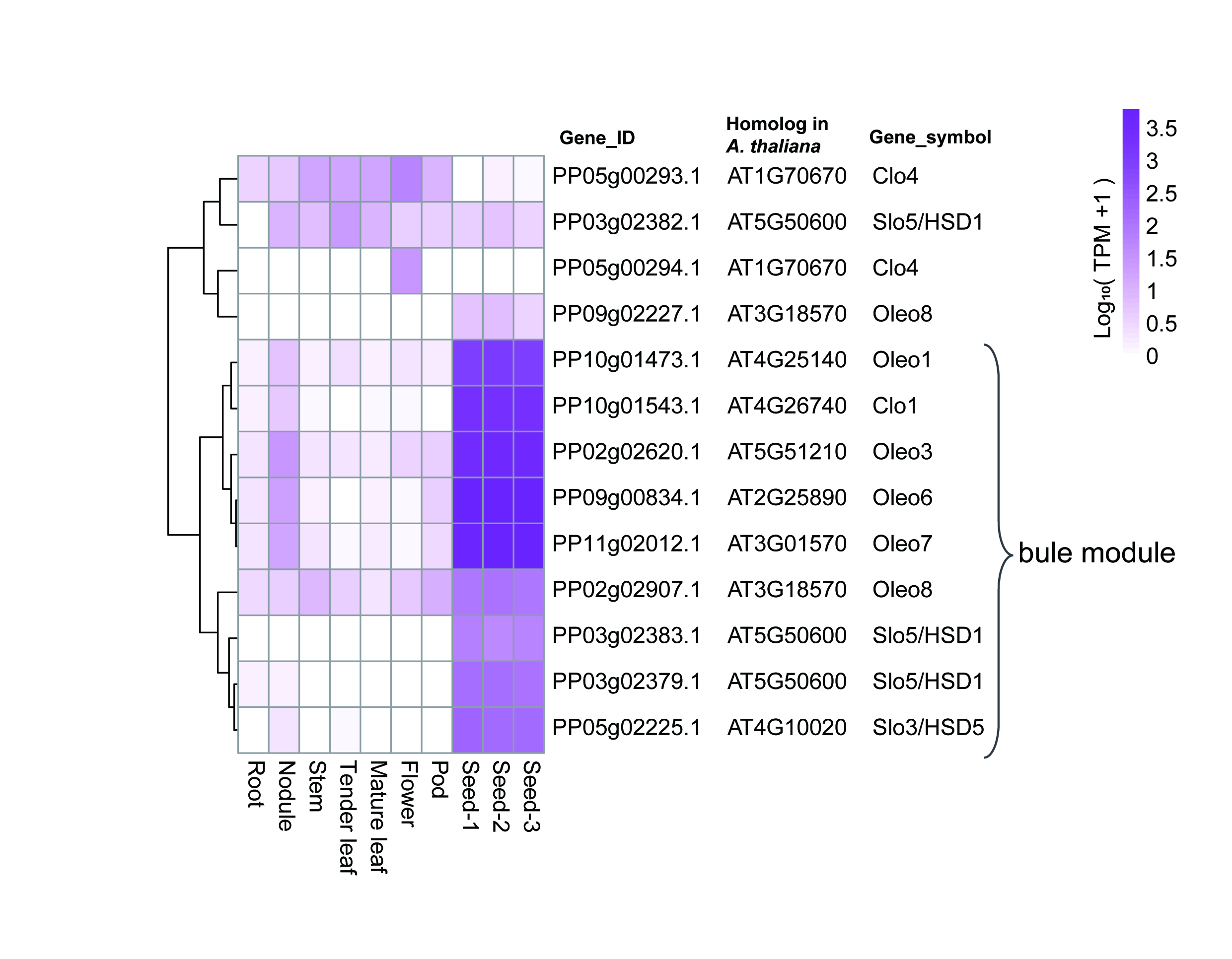

Supplement: Supplementary file 2 — Additional file 2: Figure S1. Expression heatmap of three gene families encoding lipid-body-membrane proteins (oleosin, caleosin, and steroleosin), with nine members found to belong to the seed-related module of WGCNA. [file 13068_2024_2538_MOESM2_ESM.tiff]
